# Collagen orientation probed by polarized Raman spectra can serve as differential diagnosis indicator between different grades of meniscus degeneration

**DOI:** 10.1038/s41598-021-99569-2

**Published:** 2021-10-13

**Authors:** Nikoletta Prokopi, Konstantinos S. Andrikopoulos, Amaia Soto Beobide, George A. Voyiatzis, Dionysios J. Papachristou

**Affiliations:** 1 Unit of Bone and Soft Tissue Studies, Department of Histology, University of Patras, School of Medicine, 26504 Patras, Greece; 2 Foundation for Research and Technology-Hellas, Institute of Chemical Engineering Sciences (FORTH/ICE-HT), Stadiou Str., 26504 Rio-Patras, Greece; 3 Department of Civil Engineering, University of Peloponnese, 26334 Patras, Greece; 4 Department of Pathology, University of Pittsburgh, School of Medicine, Pittsburgh, PA USA

**Keywords:** Orthopaedics, Supramolecular assembly, Diagnostic markers, Raman spectroscopy

## Abstract

The purpose of the present study was to analyze normal and degenerated menisci with Raman methodology on thin sections of formalin fixed paraffin embedding tissues and to correlate the Raman findings with the grade of meniscus degeneration. Menisci (n = 27) were removed from human knee joints after total knee replacement or meniscectomy. Following routine histopathological analysis to determine the grade of meniscal lesions obtained from healthy and degenerated formaline fixed paraffin embedded (FFPE) meniscal sections, Raman polarization approach was applied to evaluate the orientation of collagen fibrils in different levels of the same 5 μm thick FFPE meniscal tissue sections, used for histopathological assessment. We collected Raman spectra in two different polarization geometries, v-HH and v-VV, and calculated the mean value of the v-HH/v-VV intensity ratio of two Raman bands, sensitive and non-sensitive to the molecular orientation. The collagen specific amide I band at 1665 cm^−1^, has the higher sensitivity dependence on the Raman polarization. The mean values of ratio v-HH/v-VV of the 1665 cm^−1^ peak intensity was significantly higher in healthy, mean ± SD: 2.56 ± 0.46, compared to degenerated menisci, mean ± SD: 1.85 ± 0.42 (p = 0.0014). The mean values of v-HH/v-VV intensity ratio were 2.18 and 1.50 for low and high degenerated menisci, respectively (p < 0.0001). The difference of peak intensities in the two laser polarizations is decreased in the degenerated meniscus; this difference is diminishing as the degeneration increases. The v-HH/v-VV ratio was also of significant difference in low as compared to control and high grade meniscus lesions (p = 0.036 and p < 0.0001, respectively) offering valuable information for the approach of its biology and function. In the present study we showed that the 5 μm thick sections can be used for Raman analysis of meniscal tissue with great reliability, in terms of sensitivity, specificity, false-negative and false-positive results. Our data introduce the interesting hypothesis that compact portable Raman microscopy on tissue sections can be used intra-operatively for fast diagnosis and hence, accurate procedure design in the operating room.

## Introduction

The medial and lateral menisci of the knee are semicircular fibrocartilaginous tissues that play important roles in load bearing stabilization, shock absorption, joint lubrication and proprioception^1,2^. Their functions and architectural features are attributed to the well-organized network of collagen fibers interposed with meniscal cells and extracellular matrix (ECM). There are three distinct layers of collagen orientation: the superficial layer that consists of a thin layer of fibrils, the deep layer of irregularly-aligned collagen bundles and the middle layer. The latter is the larger and consists of circumferentially oriented collagen fibers^3^. Because of its unique structure, menisci transmit 50–70% of the knee loading. Injury or/and degradation increases the contact stresses in the meniscal compartments, leading to cartilage degeneration and loss and eventually subchondral bone defects^4,5^. It has been shown that resection of 15–34% of the meniscus increases contact pressure by more than 350%^6^.

Menisci are an integral part of the knee, and therefore their injuries disrupt normal knee function and mechanics, resulting in articular cartilage degeneration and thus contribute to the development of knee osteoarthritis. The most prominent molecular ECM protein of meniscus is collagen type I, which is organized into three distinct layers^3^. Menisci degeneration is characterized by the disruption of collagen fibers in the superficial and middle layers, mucinous degeneration, extracellular matrix degradation, loss of proteoglycans and chondrocyte death^7–9^.

Regrettably, such biomolecular and biochemical changes can’t be perceived by conventional methodologies such as electron microscopy and polarized light microscopy^10^. Additionally, clinical techniques such as radiography, computed tomography and magnetic resonance imaging (MRI), which are sensibility tools for early assessment of cartilage degeneration, still encounter artifacts and low resolution. Therefore the ability to detect early meniscal lesions is limited^10,11^.

Polarized Raman spectroscopy (PRS) is a vibrational spectroscopic technique that provides information about tissue biochemical composition, enabling the simultaneous analysis of mineral and organic components, molecular conformation and orientation^12^. This technique is based on the inelastic light scattering effect, in which one in a million (or even billion) photons are scattered with a different wavelength than the incident photons, following interaction with the sample. The wavelength shift is specific to the chemical bond which is vibrationally excited. By detecting all the photons, with a shifted wavelength, a Raman spectrum can be constructed. In-as-much, Raman spectroscopy creates a molecular “fingerprint” that reflects the biochemical composition of the sample^12,13^.

Raman spectroscopy has emerged as a promising tool in biomedical sciences; it is noninvasive and allows real-time detection of the lesions, can detect conformational changes in collagen structures^14^ and allows identification of molecular changes associated with osteoarthritis (OA) pathological mechanism^15^. The intensity of the Raman spectra of oriented samples depends upon the degree of molecular orientation and the polarization of incident and scattered light^16–18^. A correlation between the intensity profile of the Raman spectrum of collagen and orientation of collagen bovine tendon fiber, depending on the laser polarization has been uncovered^19^. Recent studies have used PRS for the determination of collagen fibril orientation in tendon^19–21^, bone^22–24^, articular cartilage^25^ and skin^26^. Furthermore, PRS can detect biomolecular and fiber orientation changes, associated with diseased modifications. Indeed, Ly et al. used PRS on human skin to probe the molecular modifications of the triple helix of collagen in superficial basal cell carcinoma^26^.

Triggered by the aforementioned data, in the present study we evaluated the orientation of collagen fibrils in human healthy and degenerated meniscus with PRS and compared our findings with the histologic alterations of the examined menisci.

## Materials and methods

### Specimen preparation

A total of 27 menisci, 21 lateral or medial degenerated menisci and 6 healthy meniscal tissues collected from the Orthopedic Surgery Department of 251 Hellenic Air Force (HAF) General Hospital in Athens, were included in the present study (Table 1).

**Table 1:** Data of patients: sex (*F* female, *M* male), age, grade of degeneration (*L* low grade, *H* high grade, *C* control) and respective Raman anisotropic factors defined in section “Polarized Raman spectra of healthy and degenerated menisci on FFPET tissue sections” as appropriate intensity ratios in polarized spectra.

N	Sex	Age	Degeneration grade	Raman 1/R = I_v-HH_/I_v-VV_
1	F	71	L	2.5 ± 0.4
2	F	55	L	2.5 ± 0.3
3	F	63	H	1.2 ± 0.3
4	M	54	H	1.6 ± 0.6
5	F	35	H	2.0 ± 0.1
6	F	65	H	1.6 ± 0.6
7	M	24	L	2.1 ± 0.3
8	F	57	H	1.2 ± 0.3
9	M	51	L	2.3 ± 0.5
10	F	32	L	2.2 ± 0.5
11	M	35	L	2.2 ± 0.5
12	M	38	L	2.0 ± 0.4
13	F	30	H	1.6 ± 0.4
14	F	69	H	1.6 ± 0.6
15	M	64	L	1.9 ± 0.6
16	M	84	H	1.6 ± 0.4
17	M	39	H	1.4 ± 0.4
18	F	74	L	2.2 ± 0.3
19	M	73	H	1.3 ± 0.3
20	M	53	L	1.7 ± 0.5
21	F	47	L	2.3 ± 0.5
22	F	26	C	2.6 ± 0.5
23	M	45	C	3.0 ± 0.5
24	M	31	C	2.4 ± 0.5
25	M	44	C	3.2 ± 0.2
26	F	22	C	1.9 ± 0.4
27	M	38	C	2.3 ± 0.5

The menisci were obtained from 10 men and 11 female with simultaneous osteoarthritis, (24–84 years old; mean age: 53 years) who underwent total knee arthroplasty or meniscectomy, between October 2015 and July 2016. Healthy menisci were obtained from 4 men and 2 female, with traumatic meniscal injury, without any previous history of knee pathology, (22–45 years old; mean age: 34 years). All samples were anonymized and the only information available was the histopathological grade of degeneration, the sex and age of the patient. The study was approved by the Ethics Committee of the University of Patras, all participants provided written informed consent before enrolling the study and the research was according to the appropriate guidelines and regulations.

After surgical removal, menisci were wrapped in TBS soaked gauze and frizzed at − 20 °C until the time of dissection. Punch biopsies were obtained from anterior, central and posterior regions^27,28^. It has been previously shown that freeze–thaw manipulation doesn’t affect significantly the mechanical, histological and biochemical properties of tissues, such as cartilage and meniscus, and therefore is suitable for studying the orientation of collagen network^29,30^. Each tissue specimen underwent one freeze–thaw cycle.

### Histopathological analysis

Meniscal specimens were fixed in 10% neutral buffered formalin for 24 h. Fixed tissue underwent wash with ascending alcohol and descending xylene solutions, before embedding in warm paraffin. For histopathological evaluation, 5-μm thick sections were obtained and then collected on poly-l-lysine coated slides, using a microtome. Next, sections were deparaffinized and stained with conventional hematoxylin and eosin stain^31–34^. Based upon standard histopathological criteria (the presence of inflammation and calcification, increased cellularity and meniscal cell clustering, development of myxoid changes and existence of stimulated perimeniscal layer composed of activated synovial cells), meniscal degeneration was graded as low-grade (LG) and high-grade (HG). Pathologist defined five representative areas on each meniscal sample examined for acquisition of Raman spectra.

### Raman experiments

#### Raman setup

For the Raman studies of formalin-fixed paraffin embedded tissue (FFPET) thin slide sections, the T-64000 (Jobin Yvon-Horiba) micro-Raman system was applied. The excitation wavelength (514.5 nm) was provided by a DPSS laser (Cobolt Fandango TMISO laser). The laser power on the sample was maintained at 1 mW and was focused on the samples by a 100 × microscope objective. Confocal measurements were compulsory as the glass-slide Raman bands interfered with the respective collagen bands. In this concept, rejection of most of the Raman signals from the microscope glass-slides (on which the thin samples’ cross-sections produced) was performed by optical components including a pinhole in the collected beam pathway. The confocal configuration enabled the collection of the inelastically scattered light from depths in the order of few μm matching the thickness of the thin cross-sections. In principle, all information collected originated practically from the entire sample thickness. The scattered beam passed through an appropriate edge filter (for the removal of the strong elastically scattered photons) and directed into the slit of the monochromator in the single spectrograph configuration. The resolution was kept constant in all experiments (~ 7 cm^−1^). Raman spectra were recorded by a 2D-CCD detector (Symphony II). The spectral range covered in the Raman measurements was ~ 800–1800 cm^−1^ i.e. the fingerprint region of cartilage tissue. Polarized measurements were accomplished by using an appropriate rotator in the incident beam and a set of polarizer and broad-band λ/2-plate in the scattered beam. Four distinct polarization geometries were thus feasible denoted by XY where X/Y are either H (Horizontal) or V (Vertical) depending on the direction of the incident/scattered light polarization on the measurement site with respect to directions of the reference lab-frame. All spectra were collected after positioning the sample under the microscope in such a way that the collagen fibers were distributed either along the horizontal or along the vertical direction of the lab-frame. For the adjustment, the deparaffinized meniscal sections were inspected through the optical microscope of the Raman setup operating in the transmitted bright light mode. Spectral calibration involved regular measurements of the Si reference sample, while system’s calibration with respect to polarization response was achieved by collection of a set of four spectra using all different polarization geometries from a CCl_4_ reference sample.

In addition to the micro-Raman spectrometer, Fourier Transform Raman (FT-Raman) also applied in order to analyze fresh bulk meniscal human tissue and compare the results with those obtained with the dispersive Raman ones collected from the deparaffinated thin cross sections. FT-Raman spectra were obtained using a Bruker (D) FRA-106/S component attached to an EQUINOX 55 spectrometer. A R510 diode pumped Nd:YAG laser at 1064 nm (with a maximum output power of 500 mW) was used for Raman excitation in a 180° scattering sample illumination module. An optical filtering reduced the Rayleigh elastic scattering and in combination with a CaF_2_ beam splitter and a high sensitivity liquid N_2_ cooled Ge-detector allowed the Raman intensities to be recorded from 50 to 3300 cm^−1^ in Stokes-shifted Raman region, all in one spectrum. Spectra were recorded with a resolution of 4 cm^−1^ and 200 scans accumulation.

#### Sample preparation and Raman measurements

First, 5-μm thick FFPET meniscal sections were placed on the poly-l-lysine coated slides. In order for the sections to be deparaffinized, a standard protocol has been employed^35^. More specifically, slides were heated in an oven at 60 °C, overnight and they were subsequently immersed as follows: (a) in hot xylene 60 °C (three washes for 15 min each), (b) in xylene at room temperature (two washes for 1 min each), (c) in 100% ethanol and xylene (two washes for 1 min each), (d) in 100% ethanol for 1 min, (e) in 95% ethanol for another one min, (f) in deionized water (two washes for 2 min each). Finally, the slides were placed in TBS (Tris-buffering Saline) for 5 min (TBS is an isotonic buffer that maintains the pH within a relatively narrow range). All above steps of deparaffinization were necessary in order to minimize the paraffin Raman bands in the collected Raman spectra.

Five to ten sets of polarized confocal Raman spectra i.e. with polarization perpendicular and parallel to the collagen axis of anisotropy from each human meniscus section (*n* = 6 for control and *n* = 21 for degenerated sections) were collected. The two different polarization geometries used for each measurement set were acquired in tandem on the same spot. Twenty repetitions of 60 s integration were the time collection parameters (overall measuring duration: 20 min). All spectra were collected after positioning the sample under the microscope in such a way that the collagen fibers were distributed either along the horizontal or along the vertical direction of the lab-frame. The sets of polarized spectra are denoted as h-VV and h-HH for the first case and v-VV and v-HH in the latter (low-case letter at the front indicates the orientation of the collagen fibers with respect to the lab-frame). The two different geometries of the collagen axis of anisotropy positioning were used as a confirmation of the anisotropy evaluation through the Raman spectra.

#### Raman data processing

The Raman spectra were baseline corrected and for each one of them the integrated intensity of the amide I band (~ 1665 cm^−1^) was calculated. The intensity ratio 1/R = I_v-HH_/I_v-VV_ of the above mentioned band for every set of polarized Raman spectra collected from each sample was extracted and was used for further analysis. Over 100 pairs of spectra in total were used for the comprehensive statistical analysis described below.

### Statistical analysis

Statistical comparisons used Student’s *t* test. Data are shown as mean ± standard deviation (mean ± s.d.). Significance level was defined at p ≤ 0.05. All analyses were performed using GraphPad Prism (version 8, GraphPad Software, San Diego, CA, USA). Sensitivity and specificity, as well as the false positive and false negative results associated with the Raman spectroscopic-tests, were extracted using the SPSS software (v27) under “descriptive statistics”.

## Results

### Histological findings

From 21 degenerated menisci, 11 specimens are characterized as LG and 10 as HG. Their histological characteristics of HG meniscal injuries were cell clustering, myxoid changes and increased cellularity. We did not observe inflammation or calcification. In LG meniscal degenerations cell clustering and mild elevation of tissue cellularity were the main histological features.

### Raman spectroscopy results

#### Raman spectroscopy on frozen meniscus tissues, and thin 5 μm tissue sections FFPET

In order to appreciate whether Raman analysis on FFPET sections is a reliable methodology to evaluate meniscal lesions, we compare the Raman results of frozen tissue and 5 μm thick sections placed on glass slides, as used in convectional histopathological analysis. We found that there weren’t significant differences in the Raman spectra between the two aforementioned methods.

**Figure 1:**
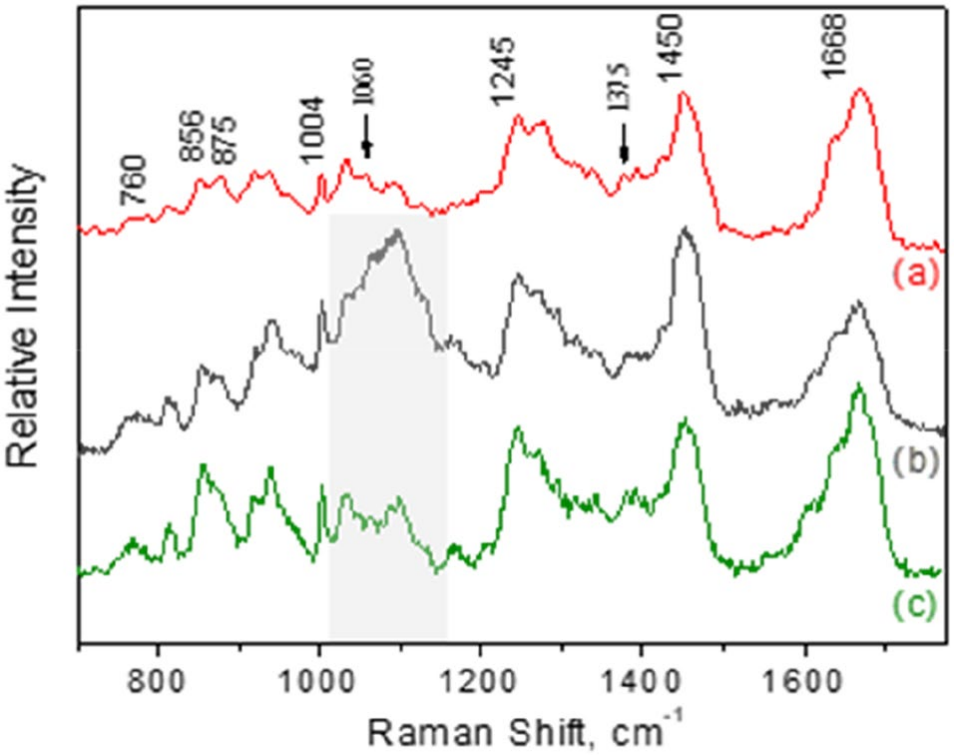
(**a**) FT-Raman spectrum (1064 nm) from fresh bulk meniscal human tissue and micro-Raman spectra (514.5 nm) from a deparaffinated thin cross section tissue using the conventional (**b**) and the confocal (**c**) configuration. The glass-slide vibrational band at ~ 1100 cm^−1^ (shadowed spectral region) is severely suppressed in the confocal spectrum.

A typical Raman spectrum (1064 nm excitation wavelength) from a frozen human meniscal tissue is depicted in Fig. 1.

The respective micro-Raman spectra (514.5 nm) from the deparaffinated thin cross sections collected from the poly-l-lysine coated slides (~ 5 μm thick) are also included in the same figure. Spectrum (b) was obtained using the conventional micro-Raman setup, while for the accumulation of spectrum (c) the confocal configuration was applied. In all depicted spectra, no specific light polarization geometry was selected. The spectral range shown is 700–1800 cm^−1^ and several bands similar to the ones found in the literature can be resolved (Table 2)^26,36–40^.

**Table 2:** Summary of major band assignment for Raman spectra of human meniscus.

Raman Shift (cm^−1^)	Assignment
760	Tryptophan ring deformation
856	Proline(C–C stretch)
875	Hydroxyproline(C–C stretch)
940	C–C backbone Collagen
1004	Phenylalanine (C–C symmetric ring stretch)
1245	Amide III (C–N stretch)
1450	CH_2_ collagen
1665	Amide I (C=O stretch) collagen

Regarding the eventual existence of GAGs, we have carefully examined the contribution of the bands around 1060 and particularly at 1375 cm^−1^, identified in all GAGs and therefore not directly related to sulphates^41^; however, their very low contribution allows no perspective to be utilized as diagnostic bands.

#### Polarized Raman spectra of healthy and degenerated menisci on FFPET tissue sections

**Figure 2:**
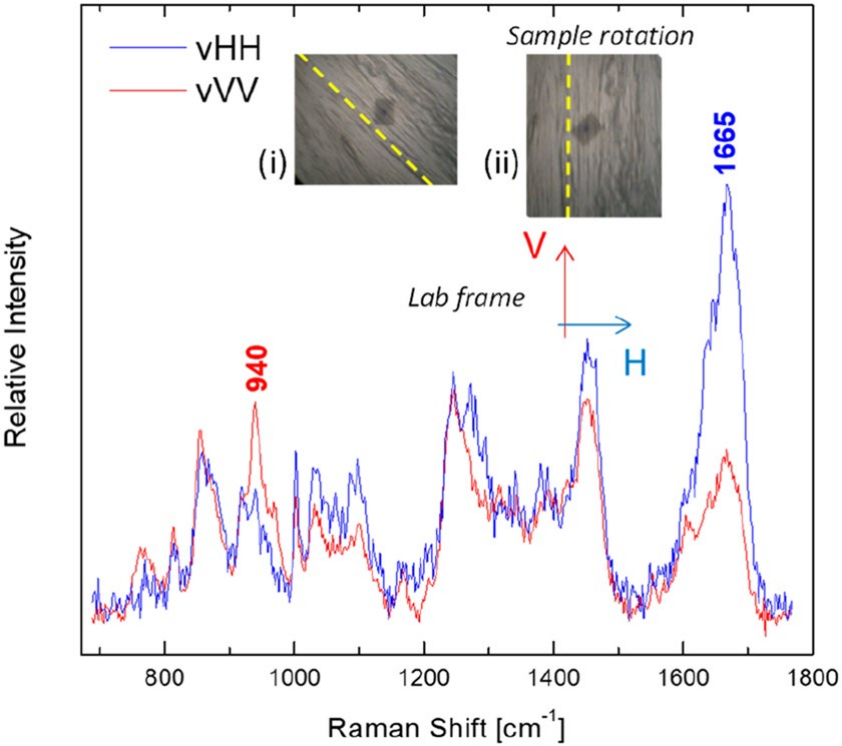
Polarized Raman spectra from the control/healthy meniscus. Inset depicts the laboratory framework directions (V and H), a micro-photograph from the meniscus cross-section (× 100 magnification), the axis of anisotropy is denoted by the dashed line (i) and the corresponding micro-photograph of the same cross-section rotated so that the axis of anisotropy is aligned with the V direction of the laboratory framework (ii).

Observation of the cross sections through the microscope attached to the Raman system enabled the identification of the collagen fibers anisotropy axis (indicated by the dashed yellow line of inset “i” in Fig. 2). In order to quantify the anisotropy, the sample had to be rotated so that the axis was aligned either towards the H or V direction of the laboratory framework (as shown in “ii”). A set of polarized Raman spectra (v-VV and v-HH) from a healthy (control) meniscus cross-section is shown in Fig. 2.

**Figure 3:**
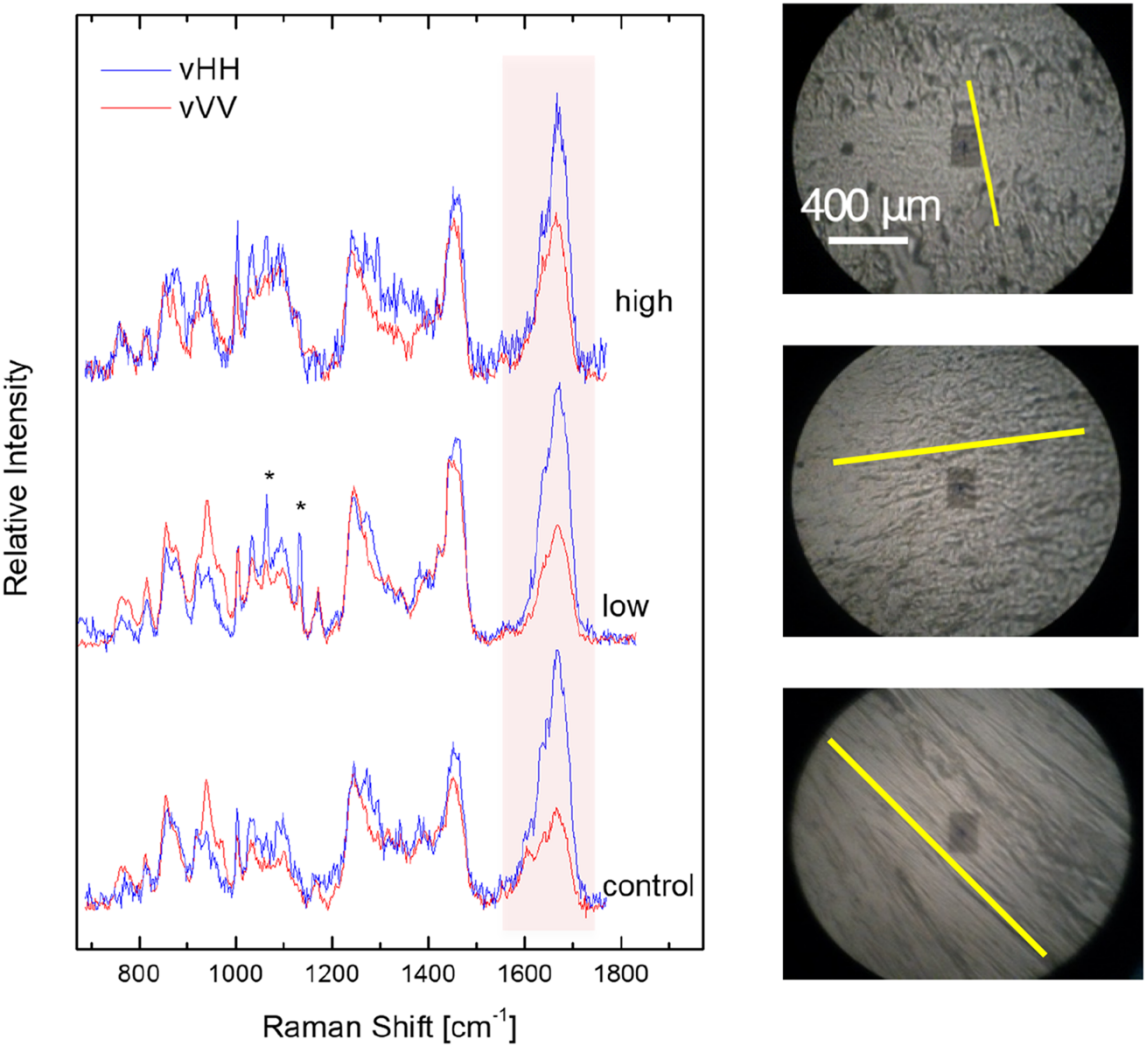
Polarized Raman spectra from the control/healthy, as well as, from menisci with low-and high-grade degeneration. Micro-photographs of the respective representative cross-sections are shown on the right. Yellow lines indicate the axis of anisotropy.

By following the same experimental procedure several sets of polarized Raman spectra were collected from all samples tested. Representative sets of spectra for the healthy and the low/high degenerated menisci are given in Fig. 3. In the same figure the corresponding micro-photographs are depicted (yellow lines designate collagen axis of anisotropy for each case).

For each set of polarized Raman spectra the integrated intensity of the 1665 cm^−1^ band was calculated and consequently the ratio 1/R = I_v-HH_/I_v-VV_ was extracted (Table 1). Average values and the respective standard deviations were determined from the measurements accomplished on healthy (6 cases) and degenerated (21 cases) menisci allowing the comparison between the two cases. The results found were 1/R = 2.56 ± 0.46 and 1.85 ± 0.42 for the healthy and degenerated menisci, respectively. The p-value calculated through the appropriate *t* test was p = 0.0014, signifying the potential of the anisotropy factor 1/R to be used as an indicator for a degenerated meniscus.

#### Anisotropy as an indicator of grade of meniscus degeneration

Our analysis uncovered that the 1/R values between LG and HG degeneration as well as among degenerated and normal meniscus were significantly different. Statistical calculations of the 1/R values revealed substantial differences between LG and HG menisci degeneration, indicated that there exists a significant difference between these two grades. Most specifically, the extracted values were (1/R)_LG-av_ = 2.18 ± 0.24 and (1/R)_HG-av_ = 1.50 ± 0.24 and (p < 0.0001). Interestingly, significant differences were found between the healthy meniscus and the meniscus with LG degeneration (p = 0.036). Table 3 shows meniscal histological grade in comparing to the mean 1/R value. The SD and the number of menisci in each group are also presented.

**Table 3:** Summary of menisci, their degeneration grades and the anisotropy average values calculated from the polarized Raman spectra. (*LG* low grade, *HG* high grade).

Menisci N	Degeneration grades	Mean 1/R = I_v-HH_/I_v-VV_	± SD
6	Control	2.56	± 0.46
11	LG	2.18	± 0.24
10	HG	1.50	± 0.24

**Figure 4:**
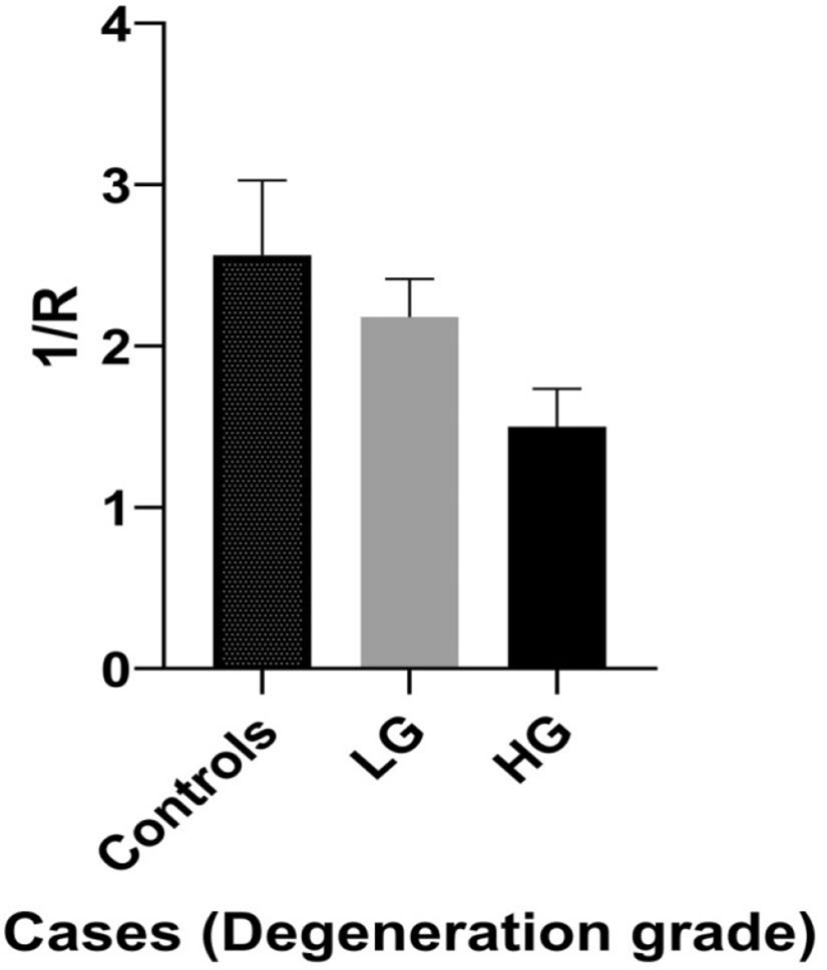
Bar chart of the 1/R mean values obtained from control menisci and menisci with LG and HG degeneration. R values correspond to the amide I band of the collagen. The differences between LG and HG as well as between LG degeneration and control menisci were remarkable (p < 0.0001 and p = 0.036 respectively).

The extracted 1/R mean values for control, LG and HG samples are depicted in the bar chart of Fig. 4. Moreover, our statistical analysis revealed that Raman could predict the presence of meniscal degeneration with a sensitivity of 83.3% and a specificity of 90.5%. Notably, Raman spectroscopy could identify all the menisci with high degeneration (as defined by histopathological analysis), with no false-positive or false-negative results. As regards menisci with low degeneration, Raman spectroscopy was associated with no false negative and 18.2% false-positive results.

## Discussion

Previous studies have shown that 10–20 μm thick FFPET sections can be used for Raman analysis, with minimal contribution from glass slide^42–44^. Stemming from this, one of the aims of the present study was to examine whether 5 μm-thick sections obtained from FFPET and used for tissues histopathological evaluation under conventional bright field microscopy, can also be used for Raman analysis. We showed that even with the use of 5 μm thick sections the contributions of slide glass is virtually zero. This is a very interesting finding, since it gives the investigator the opportunity to histologically analyze tissue sections, detect and mark the lesions and then apply Raman on the areas of interest. One additional benefit from our findings is that researchers can use FFPET archival bioptic material from Pathology Laboratories for large retrospective studies, on a huge variety of diseases, including meniscus degeneration.

Collecting Raman spectra from FFPET meniscal tissue should be as close as possible to its original/in vivo form. Therefore, in the present study we focused on the effect of tissue deparaffination and hydration on Raman procedure. Xylene deparaffinization process of thin tissue sections for Raman spectroscopy has already been used by Faolain et al. They found that spectra peaks of paraffin are reduced in intensity; nonetheless, they are present and not abolished in Raman spectra^45^. Ali et al. by applying a similar protocol, achieved complete deparaffinization of 20 μm-thick skin tissue sections. However this process affected their results by reducing the lipid content in all skin layers^44^. In the same vein, Mian et al., using 20 μm thick sections of FFPET mucous membranes, also achieved complete deparaffinization without affecting the peaks of the biochemical tissue content^43^. Several studies have documented that paraffin interferes with amide I, keratin, nucleic acid (DNA) and amide III band and prevents their expression. Peak assignments near to 1063, 1133, 1296 and to 1441 cm^−1^ are Raman contributions of paraffin due to typical C–C (carbon–carbon) stretching and CH_2_ and CH_3_ deformation. There is also a smaller contribution of peak assignment near to 1004 cm^−1^ (C–C aromatic ring), due to the small percentage of wax cycloparaffins. A worth mentioning finding of our study was that the protocol we used, resulted in a sufficient reduction of the paraffin peaks in the Raman spectra, eliminating their contribution to the peaks of interest: 1665 cm^−1^ (amide I band) and the reference peak at 1245 cm^−1^. Another notable finding was that the spectra collected from fresh tissue and deparaffinated FFPET on poly-l-lysine coated slides were similar. This indicates that the process we used did not affect the chemical characteristics of the examined tissues. Relative intensity alterations between the bands are attributed to the local anisotropic structures of collagen (see for example polarized spectra in Fig. 2). Moreover, in the present study we showed that the major Raman bands (corresponding to specific components of the sample composition) were analogous in human healthy and degenerated menisci. This finding is in line with previous studies on rabbit meniscus and articular cartilage^19,25,40,46–49^. However, we didn’t observe peaks assigned to hydroxyapatite (~ 960 cm^−1^) and calcium pyrophosphate dihydrate (CPPD) (1049 cm^−1^) crystals in either healthy or degenerated meniscus.

Previous studies have tested bone, articular cartilage and tendon with Raman methodologies. Indeed, Falgayrac et al. studied with RPS, the collagen I orientation, in sheep tendon and found that peak assignment near to 1669 cm^−1^ (amide I band) is the one most affected by laser polarization, whereas the intensity of the peak near to 1243 cm^−1^, (assigned to amide III band), remains constant. The first is due to the C=O vibrations of the peptide bond and the second to the N–H of the peptide group^23^. Others, using PRS in probing the early biochemical and orientation changes in impacted porcine cartilage, demonstrated that the significant decrease in the intensity at 1126 cm^−1^ band (pyranose ring) suggests decrease in the glycosaminoglycan content in early cartilage damage. This finding implies that PRS may be used as a tool for diagnosis and detection of early cartilage damage at the molecular level^25^. In the same vain studies on the organization of bovine articular cartilage layers applying the techniques of nanoindentation and Raman spectroscopy, showed that the functional behavior of the tissue depends mainly on the microstructure of the extracellular matrix^40^. Falgayrac et al. and Kazanci et al. studied with PRS the collagen fibers orientation and apatite crystals on human femur using ratios of spectra bands, such as the amide III band (at 1271 and 1243 cm^−1^)^23,50^. Our findings are similar to those of Kazanci et al., who reported that amide III-1245 cm^−1^ was less susceptible to polarization and therefore can be considered as peak reference^50^.

In the present work we focused on the study of collagen orientation in healthy and degenerated menisci, since the anisotropic behavior of meniscal tissue parallels its altered mechanical properties. Differences in Raman peak intensities in various layers of articular cartilage reflect a variation in the orientation of collagen fibrils rather than a change in chemical composition of the tissue. Concerning the polarized spectra of the healthy meniscus cross-sections, for some of the bands the intensity is pronounced in the v-HH geometry, while for others the favorable geometry is the v-VV. This depends on the particular vibrational mode, and more specifically, on the average orientation of the corresponding Raman tensor with respect to the axis of anisotropy. The conformation structure of the macromolecular chains results in orientation of the respective Raman tensor. For example the Raman tensor for the amide I band at ~ 1665 cm^−1^ is directed perpendicularly to the anisotropy axis (axis towards which macromolecular chains tend to align) and thus the intensity of the amide I band is enhanced when v-HH polarization geometry is used. On the other hand, the Raman tensor of the ~ 940 cm^−1^ band (assigned to the C–C vibration of collagen’s backbone) is directed along the anisotropy axis, thus, the v-VV polarization geometry results in higher intensity values. The non-dependence of the intensity of some bands (e.g. 1245 cm^−1^ of amide III) on polarization geometry is noteworthy, as they can be used as reference bands^17^.

The information provided by PRS regarding collagen orientation involves a degree of difficulty because of the relative intensity profile of tissue orientation. Levillain et al. studied the orientation of collagen in rabbit meniscal tissue, using Raman methodology, as well. Raman spectrum reception was based on the appropriate geometric cutting of the meniscus segment accordingly on perpendicular or radial orientation of collagen fibers. The experiments uncovered well-organized and dense collagen network, at the circumferential meniscus periphery, while in degenerated meniscus, the collagen network was sparse, with less compact bundles and more isolated collagen fibers^47^. In order to quantify the segmental orientation, we obtained a series of polarized Raman spectra from degenerated and healthy menisci cross-sections. Aiming at performing reliable and accurate comparisons between Raman data received from various cross-sections of both healthy and degenerated menisci, the measurement positions on the degenerated cross-sections were carefully selected to exhibit the optimum orientation profile. The recognized anisotropy axis was rotated (v or h) towards the fixed laboratory framework and the set of Raman spectra, using two different polarization geometries (VV and HH). Obviously, all comparisons made correspond to the most anisotropic areas of the degenerated meniscus cross-sections. Any randomly selected position on the degraded samples would result in greater differences of segmental orientation with respect to the control cross-sections.

The evaluation of collagen orientation within a tissue is crucial for the appreciation of tissue function and for the prediction of its biological behavior. In order to best quantify segmental orientation in macromolecular chains using Raman spectroscopy, the second and the fourth moments of the orientation distribution function denoted as P_2_ and P_4_ need to be determined. Nevertheless, the extraction of these parameters requires a number of polarized spectra, using various polarization geometries and the calculation of the intensity of a selected vibrational band for each of these geometries. In addition, precise quantification results may be particularly difficult for complex structures such as collagen. In order to extract fast semi-quantitative results and to compare samples of different anisotropies, the intensity ratio R = I_v-VV_/I_v-HH_ is frequently used^17^.

Our analysis showed that the 1/R ratio value can be used for the discrimination between normal and degenerated meniscus, as well as, between menisci with low and high degeneration. Obviously, further analysis on large number of cases and controls are required in order to substantiate our hypothesis. Nevertheless, these results encourage further investigations on human degenerated meniscus, which may reveal submolecular features associated with the pathobiology and the progression of the disease. Statistics associated with the 1/R values extracted from spectroscopic measurements may be performed if for example the values that fall within the [2.31, 3.09] range (i.e. within the standard deviation for the control samples) are characterized as positive (healthy) by the Raman spectroscopy test, while the rest are considered negative. Testing Raman values as a predictor of meniscal degeneration revealed a sensitivity of 83.3% and a specificity of 90.5%. Notably, Raman spectroscopy could identify all the menisci with high degeneration (as defined by histopathological analysis), with no false-positive or false-negative results. For the low degenerated cases Raman spectroscopy was associated with no false-negatives and 18.2% false-positive results. These results are important since menisci with high levels of degeneration cause remarkable clinical/motility problems and therefore, require fast and accurate diagnosis and treatment. Furthermore, the aforementioned findings reinforce our notion that Raman could serve as an additional tool even in the operation room. This requires the designing of a special micro-Raman setup, which will be based on parameters that consider mainly the reduction of the acquisition time; for instance, small(er) spectrograph with increased throughput (short focal length, lower dispersion power of the grating), high(er) quantum efficiency of the CCD detector with the ability of binning, dedicated/short(er) useful spectral window. Such a spectrograph could be even simpler than that we have proposed before for the on line acquisition of polarized Raman measurements in the evaluation of the molecular orientation of drawn polymers^17^. During the time passed since that particular work, new important scientific research entries immerged, which could further support the fast diagnosis using polarized Raman microscopy. The simultaneous acquisition of the polarized and depolarized Raman signal with a single detector proposed by Kiefer^51^ and the fast/quantitative single-acquisition Raman based 2D and 3D orientation mapping by simultaneous registration of multiple Raman scattering spectra obtained at different polarizations, proposed by Oleksii Ilchenko et al^52^, are among the most interesting.

## Conclusions

In the present study we showed that Raman analysis can be reliably applied on FFPET sections, used for conventional histopathological analysis and disease diagnosis. This is a notable finding since it allows the application of Raman methodology on archival tissues on a huge variety of pathological conditions, such as cancer. Moreover, herein we show that Raman analysis on FFPET sections can serve as an additional tool for the detection of medical pathologies and most importantly, for the accurate discrimination between normal and degraded menisci, as well as, between menisci with low- and high-grade degeneration.

Taken together our findings pave the way towards the development of novel, technically advanced, preferably portable Raman instruments that in combination with intra-operative histological analyses could provide faster, real time and reliable data, adding to the decision making algorithm for the management of meniscal pathologies.
